# Prevalence and risk factors for postpartum depression among women seen at Primary Health Care Centres in Damascus

**DOI:** 10.1186/s12884-019-2685-9

**Published:** 2019-12-23

**Authors:** Mayada Roumieh, Hyam Bashour, Mayada Kharouf, Salah Chaikha

**Affiliations:** 1 Department of Obstetrics and Gynecology, Faculty of Medicine, Damascus University, Damascus, Syria; 2 Department of Family and Community Medicine, Faculty of Medicine, Damascus University, Damascus, Syria

**Keywords:** Postpartum depression, Women, Prevalence, Risk factors, Syria

## Abstract

**Background:**

In Syria, there are no previous studies on postpartum depression. The aim of this study is was identify the prevalence of postpartum depression and investigate its risk factors among Syrian women seen at the Primary Health Care Centres in Damascus.

**Methods:**

This descriptive cross-sectional study was carried out between January and December 2017 in Damascus, Syria. Postpartum women seen at a convenience sample of the largest and well-utilized primary health care centres in Damascus were invited to participate in the study. The Arabic version of the validated Edinburgh Postnatal Depression Scale questionnaire was used to measure postpartum depression. A cut-off score of 13 was considered to indicate probable depression.

**Results:**

Out of a total of 1105 women participated in this study, 28.2% had a score of 13 (probable Depression). The multivariate analysis showed that postpartum depression was significantly associated with a reported a health problem during last pregnancy (OR = 2.2; 95% confidence interval [CI]: 1.4–3.5); displacement (OR = 1.4; 95% confidence interval [CI]: 1.04–1.97); perceived exposure to a lot of life stressors (OR = 5.04; 95% confidence interval [CI]: 2.4–10.5); while antenatal care had a protective effect (OR = 0.52; 95% confidence interval [CI]: 0.36–0.75).

**Conclusions:**

The prevalence of postpartum depression among Syrian women in this study was relatively high, as compared to other Arab and Non-Arab countries. Displacement due to the Syrian crisis among other factors was associated with postpartum depression. Obstetricians and other professionals should be sensitized about the importance of screening for the problem for better management.

## Background

According to the World Health Organization, about 10% of pregnant women worldwide and 13% of women who have just given birth experience a mental disorder, primarily depression. Common Perinatal Mental Disorders (CPMDs) are more prevalent in low- and lower-middle-income countries; i.e. 15.6% of women in low and lower-middle-income countries experienced a mental disorder during pregnancy and 19.8% experienced a mental disorder after childbirth [1, 2].

Postpartum depression (PPD); a non-psychotic depressive disorder classified by the Diagnostic and Statistical Manual of Mental Disorders as an Episode of Major Depressive Disorder that begins within 4 weeks of childbirth [3], is a major disabling mood disorder that affects women during childbearing years. Social factors that are associated with developing PPD include stressful life events, childcare stress, and prenatal anxiety. In addition, a history of the previous episode of PPD, marital conflict, and single parenthood are also predictive of PPD [4]. Lack of social support during pregnancy, is a relatively potent risk factor for postpartum depression, particularly in the form of high levels of depressive symptomatology [4, 5]. A recent Cochrane review indicated that psychosocial and psychological interventions, delivered by well-trained non-specialist health care providers, including the provision of intensive, professionally-based postpartum home visits, telephone-based peer support, and interpersonal psychotherapy significantly reduce the number of women who develop PPD [6].

There have been many systematic reviews of studies dealing with perinatal mental disorders in women worldwide and in specific regions, including Asia and Africa [7–9]. However, studies on PPD in the Middle East and the Arab World are scarce and have many methodological limitations. For instance, the literature review carried out by Hague at al [10]. of studies in the Middle East and the Arab World revealed that there was a great variation in the prevalence of PPD among the studied women ranging from 10 to 51.8%. Such variation in prevalence was possibly explained by the methodological limitations and differences in the design of the reviewed studies, which made comparison in the prevalence of PPD across different studies and consequently different regions almost impossible. Methodological limitations of the studies were noted as a possible explanation of the variation in prevalence [10]. For example, 51.8% PPD prevalence in Egypt is very high followed by 43% in West Bank and 41% in Iran. Studies such as that in Tunisia [11] and Oman [12] used prospective design while others used cross sectional designs.

Syria, a Middle Eastern country that experienced a major humanitarian crisis in recent years, has no previous studies on PPD. However, the mental health of Syrian people is worrisome according to the World Health Organization [13]. Syrian women have high fertility rates and they have always faced major health challenges including the low uptake of postnatal care [14, 15] though the coverage with antenatal care was very high in the country prior to war [14].

This study was conducted to investigate the prevalence of postpartum depression among Syrian women seen at primary health care centers in Damascus and identify associated risk factors.

## Methods

### Design

This is a descriptive cross sectional study that was carried out in Damascus city, the capital, between January and December 2017. Women seen at eight largest primary health care centres in Damascus representing the eight health districts in the city. The largest and the most utilized primary health care centre in each district was targeted based on statistical data shared by the health directorate, noting that some primary health care centres were out of coverage due to the current conflict [16].

### Participants

All women who recently gave birth (30–45 days postnatal) seen at the selected primary health care centres were recruited for this study. Sample size was determined based on an estimated prevalence of depressive symptoms amongst women in neighboring countries at anywhere from 15% onwards. Minimum required sample size was calculated using the prevalence formula then we allowed for design effect. An estimated sample of 1200 women was aimed at.

### Data collection

The Arabic version of the well-known validated Edinburgh Postnatal Depression Scale questionnaire [17–19] was used to measure postpartum depression. The Edinburgh Postnatal Depression Scale (EPDS) was translated and validated in Arabic [20] and is widely used in the Arab world. A cut-off score of 13 was considered to indicate probable depression. A specific questionnaire was also designed for the purpose of this study to collect data on women’s characteristics and other potential risk factors. The English version of the questionnaire can be viewed in Additional file 1.

Three trained nurses/midwives at each primary health centre collected the data. The three nurses were recruited from the family planning clinics, the vaccination clinic as well as the internal medicine clinic (one each). Those clinics were used for recruitment purposes as postpartum women are expected to be seen there. The distribution of the women between the eight PHC centres was nearly even except for one centre that underwent reconstruction work.

### Data management

Data from the field were checked for quality and entered using Excel. Data were then analyzed using the Statistical Package or the Social Sciences (SPSS), Version 22 (IBM, Chicago, Illinois, USA). All variables were subjected to univariate and bivariate analysis to determine associations between postpartum depression and women’s characteristics. To determine the main factors associated with postpartum depression and to allow for confounders, we conducted a multivariate analysis using logistic regression where we included in the model variables that showed significant associations with postpartum depression in the bivariate analysis at the *P* = 0.050 level.

### Ethical considerations

All women gave written informed consent prior to their inclusion in the study. Women with high scores on EPDS or suicidal ideation were referred for a follow up at a specialist mental health clinic through the health centre.

The Ethics Review Committee at the Faculty of Medicine, Damascus University approved the study. The approval of Ministry of Health to access the primary Health Care centres was also obtained.

## Results

### Characteristics of participants

A total of 1105 women were recruited for this study. Five hundreds and ten women were recruited from the family planning clinics at the PHC centres (46.5%) while 458 women came from vaccination clinics (41.7%) and the rest were seen at other clinic.

Table 1 shows the socio demographic characteristics of the women. The mean age was 27.7 years (SD = 6). The percentage with high education was 18.6% and those who work outside the house was 17.7%. Forty-eight percent did not live in a separate house and 11.8% shared the house with three families or more, and of importance to note that 46.5% of the women reported that they were internally displaced (forced or obliged to leave their homes as a result of the current conflict in the country).

**Table 1:** The socio-demographic characteristics of the study population

		No	%
Mean Age (SD)		27.7 (6)	
Mean Age at Marriage (SD)		21 (4.1)	
Women’s Education	Illiterate/R Or W	39	3.6
	Basic	515	47
	Secondary	337	30.8
	High	204	18.6
Women’s work status	No	910	82.3
	Yes	195	17.7
Husband’s Education	Basic or illiterate	554	50.9
	Secondary/High	534	49.1
Husband’s work	No	30	2.7
	Yes	1060	97.2
Lives in a separate house	Yes	586	53
	No	519	47
No. of families sharing the house	1	580	52.7
	2	390	35.5
	3 or more	130	11.8
Internal displacement due to war	Yes	505	46.5
	No	580	53.5

Table 2 shows the obstetric history and last pregnancy-related care of the women in the study. 33.1% were primigravida, 53.9% reported a delivery by C-section and 16.3% reported a health problem during pregnancy.

**Table 2:** Obstetric history and last pregnancy-related care of the women

	No.	%
Primigravida	365	33.1
Premature delivery	15	1.4
Delivery at Hospital (mainly public)	986	98.9
Delivered by C-Section	594	53.9
Female care provider attended the delivery	645	58.4
Received 5 or more antenatal visits during last pregnancy	860	79
Reported a health problem/complication during pregnancy	180	16.3
Reported a complicated delivery	79	7.2
Woman’s perceived pregnancy as normal	1021	92.5
Woman’s perceived delivery as normal	937	85

As shown in Table 3, 10.9% of the women reported a mental health during last pregnancy and 28.9% perceived that they were exposed to many life stressors, however 61.2% felt that were supported by family members during postpartum period.

**Table 3:** The reported and perceived health status of the women

	No.	%
Reported the existence of a chronic problem	61	5.6
Reported a mental health problem (lifetime)	101	9.2
Reported a mental health problem (last pregnancy)	119	10.9
Perceived exposure to a lot of life stressors/pressure	318	28.9
Feeling supported during postpartum period	674	61.2

### Prevalence and risk factors

This study showed that 28.2% (312 women) had a score of 13 or more on the EPDS, which refers to probable depression, while 229 women (20.7%) scored 10–12 on the EPDS, which refers to possible depression. Only seven women had the thought of harming themselves with suicidal thoughts (EPDS, question 10).

The bivariate analysis indicated that postpartum depression was significantly associated with demographic factors (younger age at marriage), socio-economic factors (women and husbands’ lower level of education), pregnancy-related factors (poor antenatal care; delivery at public hospital), conflict-related factors (displacement; multiple families at house; perceived exposure to a lot of life stressors/pressure), and health-related factors, including a reported health problem during pregnancy; reported a mental health problem; and having a newborn with health problems).

Logistic regression analysis (Table 4) revealed that postpartum depression was significantly associated with a reported a health problem during last pregnancy (OR = 2.2; 95% confidence interval [CI]: 1.4–3.5); displacement (OR = 1.4; 95% confidence interval [CI]: 1.04–1.97); perceived exposure to a lot of life stressors (OR = 5.04; 95% confidence interval [CI]: 2.4–10.5); while antenatal care had a protective effect (OR = 0.52; 95% confidence interval [CI]: 0.36–0.75).

**Table 4:** Logistic regression analysis of risk factors for postpartum depression among the women under study

		Proportion with Probable depression (No,%)	OR	95% CI
Reported a health problem during pregnancy	Yes	93, 51.7%	2.2	1.4–3.5
	No	218, 23.6%	1 (Ref)	
Received Antenatal care (5 visits or more)	Yes	219, 25.5%	0.52	0.36–0.75
	No	89, 39%	1 (Ref)	
Being Displaced from her residence	Yes	178, 35.2%	1.4	1.04–1.97
	No	128, 22.1%	1 (Ref)	
Perceived exposure to a lot of life stressors/pressure	Yes	160, 50.3%	5.04	2.4–10.5
	As usual	150, 20.6%	2.03	0.99–4.1
	No at all	10, 9.8%	1 (Ref)	

## Discussion

This study reported, for the first time in Syria, the prevalence of postpartum depression among a sample of Syrian women who lived in Damascus in 2017. The prevalence of probable depression was 28.2% using the 13 cut off point. This prevalence is higher than that reported in developed countries and some Arab countries [10]. The one recent study that reported similar prevalence was conducted in Bethlehem, Palestine [21].

It is important to note the main determinants of postpartum depression in our study were a reported health problem during last pregnancy, perceived exposure to a lot of life stressors as well as displacement being a conflict-related risk factors. Interestingly, those who reported utilizing antenatal care reported nearly 50% reduction in the prevalence of postpartum depression.

Immigrants face unique and multiple layers of challenges that may compromise their mental health and prevent them from receiving adequate and equitable care. For immigrant women, many of the stressors are especially compounded in the vulnerable postpartum period, resulting in PPD [22, 23]. Maternal depression was an important feature in Syrian refugee women recently resettled in Canada, in a mixed methods research design included 12 Syrian refugee women who migrated to Saskatoon in 2015–16 [24]. Thus, in our study, the issue of internal displacement as an important determinant of postpartum depression, indicate that more research is needed as to define the psychological, social and services variables related it; that could result in depressive symptoms. The number of internally displaced population in the country was over 6 million according to UNHCR [25].

The main limitations of this study include the issue of design which was not prospective and further that the recruitment was only from women who are using the primary health care centres.

## Conclusion

The findings of this study should alert Syrian health professionals that postpartum depression needs to be screened for in the usual antenatal and postnatal care of Syrian women. This study indicated that nearly one of three women have postpartum depression, this means that the burden of mental health in Syria is huge. This burden is not only on women but also on their children and families. Our findings are concerning, recognizing that the promotion of mental health and wellbeing is among health priorities of the global development agenda, as Target 3.4 of the Sustainable Development Agenda requests that countries: “By 2030, reduce by one third premature mortality from non-communicable diseases through prevention and treatment and promote mental health and well-being.”.

## Supplementary information

**Additional file 1.** The research instrument in English.

## Data Availability

All materials and dataset for this study are available from the corresponding author on reasonable request and approval of all co-authors.

## Abbreviations

CI: Confidence interval
CPMD: Common Perinatal Mental Disorders
EPDS: Edinburgh Postnatal Depression Scale
OR: Odds ratio
PPD: Postpartum depression
WHO: World Health Organization
