# Supplementary material for: Prevalence and risk factors for postpartum depression among women seen at Primary Health Care Centres in Damascus
Source: BMC Pregnancy Childbirth. 2019 Dec 23;19:519. doi: 10.1186/s12884-019-2685-9 (PMC6929307; doi:10.1186/s12884-019-2685-9)
Supplement: Supplementary file 1 — Additional file 1. The research instrument in English. [file 12884_2019_2685_MOESM1_ESM.docx]

**Additional file 1: The research instrument in English**

**Damascus University**

**Faculty of Medicine**

**Study on prevalence of Postnatal depression among women seen at Primary Health Care Centres in Damascus**

**Section One:** General Questionnaire

A: General information

| 1. Data collector code |  |
| --- | --- |
| 1. Code for the Health centre |  |
| 1. Date of Interview | ------ |
| 1. Serial No | --- |
| 1. Pace where interview took place: 1- Family Planning Clinic 2-Vaccination Clinic |  |

B: Women and family Characteristics

| 1. Woman age in Years |  |
| --- | --- |
| 1. Woman’s status of work: 1-Does not work 2-has not worked during pregnancy 3- has worked during pregnancy |  |
| 1. What is the job of the woman?------------- |  |
| 1. Woman’s education status: 1- Illiterate 2- Elementary 3-Secondary 4- High Education |  |
| 1. Husband’s status of work: 1-Does not work 2-is working 3- Does not exist |  |
| 1. What is the job of the husband?------------- |  |
| 1. Husband’s education status: 1- Illiterate 2- Elementary 3-Secondary 4- High Education |  |
| 1. Type of House 1- 2- Shared |  |
| 1. No. of families sharing the house |  |
| 1. Address of the house |  |
| 1. was the woman oblihed to change her accondation due to current situation |  |

C: Obstetrical history

| 1. Age at marriage |  |
| --- | --- |
| 1. DOB |  |
| 1. No. of days between delivery date and interview |  |
| 1. Parity: 1- nulliparous 2- multiparous |  |
| 1. Age of pregnancy: 1-full term 2- less than 36 weeks |  |
| 1. Place of delivery: 1- Private hospital 2- Public hospital 3- Home 4- Other, specify ….. |  |
| 1. Type of Delivery: 1- Normal 2-C section 3- Instrumental |  |
| 1. What is the sex of the birth attendant? 1- Male 2- Female |  |
| 1. Sex of the baby: 1- Male 2- Female |  |
| 1. No. of newborns: 1- single 2- twin 3- three or more |  |
| 1. Order of the baby in the family |  |
| 1. Health status of the newborn at birth: 1- good 2-has health problems |  |
| 1. What is the health problem |  |
| 1. No of antenatal visits: 1- 5 visits or more 2- less than 5 visits |  |
| 1. Has the woman suffered of health problem during pregnancy? 1- Yes (please specify…..2-No |  |
| 1. Has the woman suffered of a complicated deliver 1- Yes (please specify…..2-No |  |
| 1. Has the woman breastfed? 1- Yes 2- No |  |
| 1. How has the woman perceived her pregnancy? 1-Normal 2- Abnormal |  |
| 1. How has the woman perceived her delivery? 1-Normal 2- Abnormal |  |

D: Mental health of the woman

| 1. Is the woman suffering of a diagnosed chronic health problem: 1- No 2- yes for one Disease (Specify…. ), 3- More than one disease |  |
| --- | --- |
| 1. Has the woman been diagnosed with a mental health problem in her lifetime? 1- yes 2-No |  |
| 1. Has the woman been diagnosed with a mental health problem while pregnant? 1- Yes 2-No |  |
| 1. Did the woman feel supported after her delivery? 1-mainly by one person (Specify….) 2- None 3- supported by more than one person |  |
| 1. How does the woman evaluate her life 1- Very stressful 2- normal 3- Not stressful |  |
| 1. Note the score of EPDS here |  |

**Section TWO:** EPDS Questionnaire (Original)

As you have recently had a baby, we would like to know how you are feeling. Please NOTE the answer, which comes closest to how you have felt IN THE PAST 7 DAYS, not just how you feel today.

Here is an example, already completed.

I have felt happy:

Yes, all the time

Yes, most of the time

No, not very often

No, not at all

This would mean: “I have felt happy most of the time” during the past week.

Please complete the other questions with me in the same way.

**In the past 7 days:**

| EPDS1 | I have been able to laugh and see the funny side of Things | As much as I always could |
| --- | --- | --- |
|  |  | Not quite so much now |
|  |  | Definitely not so much now |
|  |  | Not at all |
| EPDS2 | I have looked forward with enjoyment to things | As much as I ever did |
|  |  | Rather less than I used to |
|  |  | Definitely less than I used to |
|  |  | Hardly at all |
| EPDS3 | I have blamed myself unnecessarily when things went wrong | Yes, most of the time |
|  |  | Yes, some of the time |
|  |  | Not very often |
|  |  | No, never |
| EPDS4 | I have been anxious or worried for no good reason | No, not at all |
|  |  | Hardly ever |
|  |  | Yes, sometimes |
|  |  | Yes, very often |
| EPDS5 | I have felt scared or panicky for no very good reason | Yes, quite a lot |
|  |  | Yes, sometimes |
|  |  | No, not much |
|  |  | No, not at all |
| EPDS6 | Things have been getting on top of me | Yes, most of the time I haven’t been able to cope at all |
|  |  | Yes, sometimes I haven’t been coping as well as usual |
|  |  | No, most of the time I have coped quite well |
|  |  | No, have been coping as well as ever |
| EPDS7 | I have been so unhappy that I have had difficulty sleeping | Yes, most of the time |
|  |  | Yes, sometimes |
|  |  | Not very often |
|  |  | No, not at all |
| EPDS8 | I have felt sad or miserable | Yes, most of the time |
|  |  | Yes, quite often |
|  |  | Not very often |
|  |  | No, not at all |
| EPDS9 | I have been so unhappy that I have been crying | Yes, most of the time |
|  |  | Yes, quite often |
|  |  | Only occasionally |
|  |  | No, never |
| EPDS10 | The thought of harming myself has occurred to me | Yes, quite often |
|  |  | Sometimes |
|  |  | Hardly ever |
|  |  | Never |
